# Comparative genomic analyses of the clinically-derived *Winkia* strain NY0527: the reassignment of *W. neuii* subsp. *neuii* and *W. neuii* subsp. *antitratus* into two separate species and insights into their virulence characteristics

**DOI:** 10.3389/fmicb.2023.1147469

**Published:** 2023-04-21

**Authors:** Xunchao Cai, Yao Peng, Meng Li, Yifeng Qiu, Yuhan Wang, Long Xu, Qi Hou

**Affiliations:** ^1^Department of Gastroenterology and Hepatology, Shenzhen University General Hospital, Shenzhen University, Shenzhen, China; ^2^Department of Laboratory Medicine, Shenzhen University General Hospital, Shenzhen University, Shenzhen, China; ^3^Department of Urology, Shenzhen University General Hospital, Shenzhen University, Shenzhen, China; ^4^International Cancer Center, Shenzhen Key Laboratory, Hematology Institution of Shenzhen University, Shenzhen, China

**Keywords:** abscess, *Winkia neuii*, bacterial infections, complete genome, virulence traits

## Abstract

**Background:**

*Winkia neuii*, previously known as *Actinomyces neuii*, is increasingly recognized as a causative agent of various human infections, while its taxonomy and genomic insights are still understudied.

**Methods:**

A *Winkia* strain NY0527 was isolated from the hip abscess of a patient, and its antibiotic susceptibility was assessed. The genome was hybrid assembled from long-reads and short-reads sequencing. Whole-genome-based analyses on taxa assignment, strain diversity, and pathogenesis were conducted.

**Results:**

The strain was found to be highly susceptible to beta-lactam antibiotics, but resistant to erythromycin, clindamycin, and amikacin. The complete genome sequences of this strain were assembled and found to consist of a circular chromosome and a circular plasmid. Sequence alignment to the NCBI-nt database revealed that the plasmid had high sequence identity (>90%) to four *Corynebacterium* plasmids, with 40–50% query sequence coverage. Furthermore, the plasmid was discovered to possibly originate from the sequence recombination events of two *Corynebacterium* plasmid families. Phylogenomic tree and genomic average nucleotide identity analyses indicated that many *Winkia* sp. strains were still erroneously assigned as *Actinomyces* sp. strains, and the documented subspecies within *W. neuii* should be reclassified as two separate species (i.e., *W. neuii* and *W. anitratus*). The core genome of each species carried a chromosome-coded beta-lactamase expression repressor gene, which may account for their broadly observed susceptibility to beta-lactam antibiotics in clinical settings. Additionally, an *ermX* gene that expresses fluoroquinolone resistance was shared by some *W. neuii* and *W. anitratus* strains, possibly acquired by IS6 transposase-directed gene transfer events. In contrast, tetracycline resistance genes were exclusively carried by *W. neuii* strains. In particular, *W. neuii* was found to be more pathogenic than *W. anitratus* by encoding more virulence factors (i.e., 35–38 in *W. neuii* vs 27–31 in *W. anitratus*). Moreover, both species encoded two core pathogenic virulence factors, namely hemolysin and sialidase, which may facilitate their infections by expressing poreformation, adhesion, and immunoglobulin deglycosylation activities.

**Conclusion:**

This study highlights the underappreciated taxonomic diversity of *Winkia* spp. and provides populational genomic insights into their antibiotic susceptibility and pathogenesis for the first time, which could be helpful in the clinical diagnosis and treatment of *Winkia* spp. infections.

## 1. Introduction

*Winkia neuii*, previously known as *Actinomyces neuii*, are facultatively anaerobic, Gram-positive, asporogenous, and catalase-positive organisms that are frequently isolated from clinical samples (Yang and Grant, 2019). The initial isolation of this species was reported in 1985 by Coudron et al. from the vitreous fluid of patients with endophthalmitis (Coudron et al., 1985). *W. neuii* isolates were classified as CDC (Centers for Disease Control) fermentative coryneform group 1 or group 1-like species in the 1980s until they were placed in the genus *Actinomyces* in 1994, based on a combination of 16S rRNA sequence identity, cellular and metabolic fatty acid profiles, and DNA–DNA hybridization ratio (Funke et al., 1993, 1994). Subsequently, several researchers have proposed the creation of a novel genus for *A. neuii*, as it exhibited a sequence similarity and chemotaxonomy closer to the genera *Varibaculum* and *Mobiluncus* than to *Actinomyces bovis* (Schaal et al., 1999; Hoyles et al., 2004). However, only recently in 2018, *A. neuii* was separated from the genus *Actinomyces* and was reassigned as *W. neuii*, and it is validly published as the sole species of the genus *Winkia* under the ICNP (International Code of Nomenclature of Prokaryotes) rule (Nouioui et al., 2018). Within the species, two subspecies, *W. neuii* subsp. *neuii* and *W. neuii* subsp. *anitratus* (anitrata means not reducing nitrate), have been assigned based on their biochemical activities and 16S rRNA sequence identities (Nouioui et al., 2018).

*W. neuii* has been implicated in hundreds of cases of human infection since its first isolation (Gómez-Garcés et al., 2010; Zelyas et al., 2016). The most prevalent types of infections caused by *W. neuii* are abscesses and infected atheroma, followed by infected skin lesions, urinary tract infections, endophthalmitis, and bacteremia, including endocarditis (Funke and Von Graevenitz, 1995; Roustan et al., 2009; Gómez-Garcés et al., 2010; von Graevenitz, 2011; Könönen and Wade, 2015; Yang and Grant, 2019; Giannoulopoulos and Errington, 2022). In rare cases, *W. neuii* infections cause premature labor and neonatal sepsis (Alsohime et al., 2019), and even primary actinomycosis (Leenstra et al., 2017). Previous studies have shown that *W. neuii* strains take a proportion of 8–17% in the clinically isolated *Actinomyces* spp. (Gómez-Garcés et al., 2010; Barberis et al., 2017). Researchers also discovered that the most common body sites for *W. neuii* colonization are the mammary, axillary, and inguinal areas (Hall, 2008), as well as the blood, foreign body devices, urinary tract, infected joints, and soft tissues (von Graevenitz, 2011; Leal Jr et al., 2016; Chen et al., 2020; Hara et al., 2021). To the best of our knowledge, no *W. neuii* has been isolated from environmental samples, therefore, infections caused by *W. neuii* were believed to be endogenous (von Graevenitz, 2011; Graffi et al., 2012). Fortunately, favorable outcomes are typically achieved through surgical removal of the infected focus (von Graevenitz, 2011), or using antibiotic treatment, as the *W. neuii* strains are susceptible to a wide range of antibiotics, including penicillin, ampicillin, the cephalosporins, imipenem, vancomycin, erythromycin, and clindamycin (Mann et al., 2002; Watkins et al., 2008; von Graevenitz, 2011). However, the genomes of most *W. neuii* isolates have not been sequenced, and the pathogenetic mechanisms and virulence traits of this organism remain unclear (Zelyas et al., 2016). Furthermore, due to the similarity of morphology and biochemical characteristics between *Actinomyces* and *Corynebacterium*, isolates from *W. neuii* could be wrongly assigned, and the occurrence and diversity of this organism in clinical specimens may be underestimated (Zelyas et al., 2016).

In recent years, the widespread use of matrix-assisted laser desorption ionization time-off-flight mass spectrometry (MALDI-TOF MS) in clinical laboratories has greatly increased the accuracy of identifying bacteria that were previously difficult to identify (Könönen and Wade, 2015), and it has been successfully applied to identify *W. neuii* in clinical practices (De Vreese and Verhaegen, 2013). However, manufacturers of MALDI-TOF MS targeted mostly clinically important microorganisms and provided relevant reference spectra in the databases, which may make this method fail to identify novel, highly similar and/or rare-isolated species that lack documented spectra data (Rahi et al., 2016; Ha et al., 2019; Welker et al., 2019). Nevertheless, the creation of open-source databases that incorporate MALDI-TOF MS spectra of all known species and keep them updated is now a great challenge (Rahi et al., 2016; Chen et al., 2021). The development of next-generation sequencing technology has facilitated the opportunity to obtain the whole genome of clinical isolates at low cost, which is helpful for researchers to assign microbial taxonomy at strain level, with a much higher resolution than that of MALDI-TOF MS. Moreover, the genomic sequences obtained from the microorganisms are helpful in deciphering their pathogenetic mechanisms.

As of February 23rd, 2023, only seven draft-level genomes of *W. neuii* have been deposited in the NCBI Genome RefSeq database. Furthermore, their populational genomic features related to pathogenesis, such as plasmid carriage and virulence factor coding genes, have not been characterized. In this study, we isolated a *Winkia* strain NY0527 from the sanies of hip abscess in a 27-year-old male patient and *de novo* assembled the genome at the completed level using Illumina and Nanopore sequencing reads. Taxonomy assignments and comparative genome analyses based on whole genome sequences were then performed to clarify the phylogeny, general genomic features, and gene functions related to virulence, adaptation, and pathogenesis.

## 2. Methods and materials

### 2.1. Specimen collection, bacteria isolation, identification, and antimicrobial susceptibility

Sanies were sterilely collected from the hip abscess of a 27-year-old male patient during the surgery debridement procedure. The specimen was then transferred to the clinical microbiology laboratory for culturing. Specimen was streaked onto blood agar and was incubated aerobically at 35–37°C for 3 days. Colonies that appeared on the agar plates were subsequently purified through re-streaking. The colonies obtained were then identified using MALDI Biotyper RTC (Bruker Daltonics, Germany). Briefly, single colony was transferred to the 96-target spot of the MALDI Biotyper (MALDI Biotyper 3.1), and 1 μL of bacterial test standard and matrix solution were consecutively added. Identification was carried out with the default settings. Subsequently, the MALDI log-score was calculated by matching the converted spectra data of the isolate with the reference spectra presented in the database (version 4.0). Following the manufacturer’s instructions, a log-score more than or equal to two indicates high-confidence identification at the species level. Antimicrobial susceptibility testing was performed using Etest (bioMérieux, France) or disk diffusion method (K-B method) by the Department of Laboratory Medicine, Shenzhen University General Hospital, Shenzhen University, in accordance with the Clinical and Laboratory Standards Institute (CLSI) reference method (Wayne, 2011). The antimicrobial susceptibility of the strain was determined by interpreting the antibiotic MICs from Etest and the inhibitory zone diameter from K-B, using the breakpoints established by Clinical and Laboratory Standards Institute (CLSI) (2003).

### 2.2. Genomic DNA extraction, sequencing, and genome assembly

The bacterial colony identified by the MALDI Biotyper was transferred and completely dispersed into a 1.5 ml germ-free tube with 200 μL of sterile PBS, which was then spread onto the blood agar and cultured aerobically at 35°C for 3 days. Subsequently, the bacterial cells were washed from the agar plate using 1 mL of sterile PBS and transferred into a 1.5 mL germ-free tube. Genomic DNA extraction was performed immediately using the TaKaRa MiniBEST Bacteria Genomic DNA Extraction Kit (Takara, Japan), following the manufacturer’s instructions. Genomic DNA quality was checked using the Synergy HTX Multi-Mode Reader (BioTek, United States). Genome sequencing was conducted on two platforms, namely the Nanopore PromethION platform (MAGIGENE, Guangzhou, China) and the Illumina NovaSeq platform (Novogene, Nanjing, China), to generate long reads and short reads, respectively. For the long-reads sequencing, sequencing library was prepared using the 1D Ligation Kit (SQK-LSK109, Oxford Nanopore Technologies) by following the manufacturer’s instructions. Subsequently, the sequencing was carried out on an R9.4.1 flow cell. Base calling was performed using MinKNOW v1.15.4 and low-quality reads (i.e., scores <7) were removed. The short-reads sequencing library was constructed with a 350 bp insert size and sequenced using the PE150 strategy. The genome was *de novo* assembled and in a hybrid manner using the Unicycler v0.4.9b assembler, with the default hybrid assembly pipeline (Wick et al., 2017).

### 2.3. Phylogenomic characterization, pangenome extraction, and plasmid detection

The gtdbtk_wf workflow implemented in GTDT-Tk software was used for further validation of the taxonomy assignment (Parks et al., 2018). The Similar Genome Finder implanted in PATRIC v3.6.10^2^ was employed to find public released genomes that with high sequence similarity to the assembled genome. The average nucleotide identity (ANI) between the assembled genome and the similar genomes was calculated using fastANI (Jain et al., 2018). Coding sequences (CDSs) and annotation files (e.g., gff files) from these genomes were predicted using Prokka v1.14.5 with the default parameters (Seemann, 2014). The pangenome was subsequently extracted from the resultant gff files using Roary v3.11.2 (Page et al., 2015), and CDSs that were present in over 95% of genomes were identified as the coregenome. Additionally, a phylogenomic tree of these genomes based on the whole-genome CDSs was constructed using CVTree3 with default parameters (Zuo, 2021). Plasmids were predicted using PlasForest v1.2^3^ based on machine learning from sequence homology.

### 2.4. Genome annotation and comparative genomics

Comprehensive genome function annotation was conducted using eggNOG-mapper v2.1.5, which incorporates multiple databases, including Carbohydrate-Active enZYmes (CAZy), Cluster of Orthologous Groups (COG), and Kyoto Encyclopedia of Genes and Genomes (KEGG) (Huerta-Cepas et al., 2017). Genome subsystem function (transporters, virulence factors, drug targets, antibiotic resistance genes, antimicrobial resistance genes, etc.) was annotated using the Rapid Annotation using Subsystem Technology server^4^ (Overbeek et al., 2014). Pathogenic factor genes, including virulence genes, toxin genes, and antimicrobial resistant genes (ARG), in the genomes were predicted using PathoFact v1.0 with the “complete” parameter. The results also reported the localization of these genes on mobile genetic elements (MGE) and the chromosome. Pathogenic attributes (i.e., pathogenic or non-pathogenic) of the predicted genes were determined by using the HMM model and the random forest classifier implanted in PathoFact with default settings (de Nies et al., 2021).

## 3. Results

### 3.1. Clinical presentation and diagnostic findings

A 27-year-old male patient presented with a painful lesion on his right hip, which he had developed 3 days prior to admission. He had no special medical history and no other relevant symptoms were noted. On physical examination, a painful, fluctuating, 5.0 × 4.0 cm mass with skin erythema on the right hip was detected. The ultrasound showed a mixed fluid collection measuring 4.1 × 1.5 × 2.4 cm, indicating the presence of an abscess. A peripheral hematological white blood cell count revealed 7.73 × 10^9^/L (3.5–9.5 × 10^9^), and C-reactive protein was measured at 12.04 mg/L (0–10.0). The patient was empirically treated with cefuroxime (1.5 g, q12h) and ornidazole (0.5 g, q12 h) prior to undergoing surgical debridement for his hip abscess. One day after initiation of antibiotic treatment, surgical debridement was performed, followed by a 14-day course of antibiotic therapy, which successfully cured him. A strain, designated NY0527, was isolated from the purulent fluid sample collected during the surgical operation, which was identified as *Actinomyces neuii* subsp. *anitratus* (now known as *W. neuii* subsp. *anitratus*) with high confidence by using MALDI Biotyper RTC (Supplementary Table S1). The strain exhibited susceptibility to most of the tested antibiotics, including levofloxacin, ciprofloxacin, vancomycin, meropenem, penicillin, ceftriaxone, cefepime, gentamicin and tetracyclines, among which the strain is highly susceptible to the beta-lactam antibiotics (i.e., meropenem, penicillin, ceftriaxone, and cefepime) and tetracyclines antibiotics (i.e., tetracycline) (Table 1). In contrast, this strain was resistant to only three tested antibiotics (i.e., erythromycin, clindamycin, and amikacin) (Table 1).

**Table 1:** Antibiotic susceptibility of strain NY0527.

Antibiotics^a^	Testing method^a^		Antibiotics category	Susceptibility
	Etest MICs	K-B inhibitory zone		
Levofloxacin	2 μg/mL	14 mm	fluoroquinolones	S
Ciprofloxacin	–	20 mm	fluoroquinolones	S
Erythromycin	–	8 mm	macrolides	R
Clindamycin	–	6 mm	lincosamides	R
Vancomycin	–	29 mm	glycopeptides	S
Meropenem	<0.016 μg/mL	–	beta-lactam	S
Penicillin	0.064 μg/mL	–	beta-lactam	S
Ceftriaxone	<0.016 μg/mL	–	beta-lactam	S
Cefepime	–	40 mm	beta-lactam	S
Gentamicin	–	15 mm	aminoglycosides	S
Amikacin	–	8 mm	aminoglycosides	R
Tetracycline	–	32 mm	tetracyclines	S

a The testing method Etest or K-B was determined by the Department of Laboratory Medicine of Shenzhen University General Hospital, using the routinely adopted methods in clinical testing of each antibiotics.

### 3.2. General genomic features

**Figure 1:**
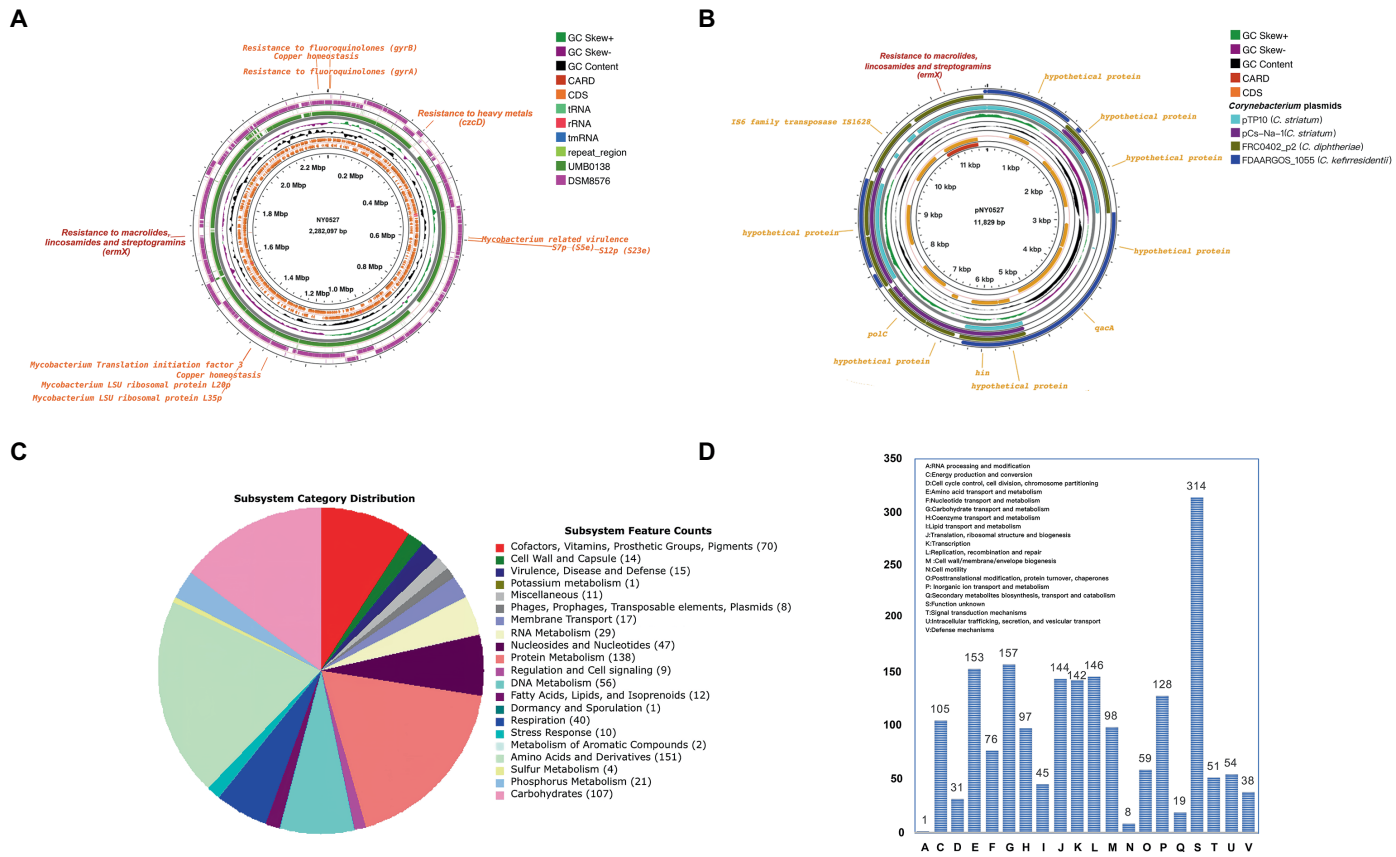
General genomic features of strain NY0527 characterized by the complete genome sequences. **(A)** The display of the circular chromosome sequence of strain NY0527. From inner to outer rings: genome backbone of strain NY0527 with ruler, antibiotic resistant genes, CDSs on the forward and reverse strands (RNAs and repeat sequence regions are displayed in the CDSs circles), GC content, GC skew, genome sequence of *Actinomyces* sp. UMB0138, genome sequence of *W. neuii* DSM8576^T^. **(B)** The display of the circular plasmid sequence pNY0527. From inner to outer rings: genome backbone of pNY0527 with ruler, antibiotic resistant genes, CDSs on the forward and reverse strands, GC content, and GC skew. The legend on the right is shared by **(A)** and **(B)**. **(C)** Genomic subsystem functions annotated using the RAST. **(D)** Genomic COG functions annotated using the eggNOG-mapper.

Two complete circular contigs free of Ns were generated from the hybrid assembly. Genome sequence quality check using checkM indicated that the assembled contigs are of high-quality (completeness = 99.84%, contamination = 0.95%, strain heterogeneity = 0.00%). The plasmid prediction analysis revealed that the larger circular contig is the chromosomal sequence, while the smaller circular contig is the plasmid sequence. In summary, strain NY0527 comprises a circular chromosome with a total length of 2,282,097 bp, 2,125 CDSs, 12 rRNAs, 48 tRNAs, and a G + C content of 56.61%; and a plasmid with a total length of 11,829 bp, 12 CDSs, and a G + C content of 56.31%. Taxon assignment using GTDB-Tk recognized strain NY0527 as a member of *Actinomyces* sp. with the ANI values of 98.83 and 81.66% to *Actinomyces* sp. UMB0138, and *W. neuii* DSM8576^T^ (DSM8576 is the type strain of *W. neuii*), respectively, which suggested that strain NY0527, as well as strain UMB0138, did not belong to the species *W. neuii*. As the MALDI Biotyper had identified strain NY0527 as *W. neuii* subsp. *antitratus* with high confidence, we can deduce from here that *W. neuii* subsp. *antitratus* should be reassigned as a separate species, rather than a subspecies of *W. neuii* (Supplementary Table S1). Genome functional annotation of NY0527 using the RAST identified several CDSs coding virulence and antibiotic or heavy metal resistant functions such as Mycobacterium-like virulence factor, fluoroquinolones resistance, macrolides/lincosamides/streptogramins resistance, and copper homeostasis. Most of these features (e.g., virulence genes and antibiotic resistant genes) were shared by the genomes of *Actinomyces* sp. UMB0138 and *W. neuii* DSM8576^T^ (Figure 1A). Nevertheless, the *ermX* gene was absent in the genome of *W. neuii* DSM8576^T^. Sequence inversions and losses were more prevalent in the genome of *W. neuii* DSM8576^T^ than that in *Actinomyces* sp. UMB0138, when aligned to the genome sequence of NY0527 (Figure 1A). No plasmid sequences were identified in the genomic sequences of *Actinomyces* sp. UMB0138 and *W. neuii* DSM8576^T^. By sequence alignment against the NCBI-nt database, we found that the complete plasmid sequence pNY0527 showed no significant sequence similarity to that of any *Actinomyces* spp. or *Winkia* spp., while it displayed high sequence similarity to the chromosomal or plasmid sequences from *Corynebacterium* spp. with the query sequence coverage of about 40–50% (Supplementary Table S2), implying the occurrence of plasmid-directed trans-genus horizontal gene transfer (HGT) events. Notably, although only 40–50% of the pNY0527 sequence was mapped to each plasmid from the three *Corynebacterium* species (i.e., two from *C. striatum*, one from *C. kefirresidentii*, and one from *C. diphtheriae*), the total fragments mapped to the *C. kefirresidentii* plasmid (i.e., FDAARGOS_1055 unnamed) and the *C. diphtheriae* plasmid (i.e., FRC0402_p2) almost completely covered the entire sequence of pNY0527, indicating that the pNY0527 might have originated from the sequence recombination events of these plasmid families (Figure 1B). The pNY0527 carries an *ermX* gene flanked by IS6 family transposes (IS628), and this gene array was also partially or entirely presented in the *C. striatum* plasmid pTP10 and the *C. diphtheriae* plasmid FRC0402_p2 (Figure 1B). The transposes within this gene array may have facilitated the transfer of the *ermX* gene from the plasmid to the chromosome in strain NY0527 or in the *Corynebacterium* spp. Moreover, pNY0527 carries *qacA* that encodes efflux-mediated antiseptic (intercalating dyes and other organic cations) resistance proteins; and carries *hin* that encodes DNA invertase, which can regulate flagellar phase variations in pathogens (Brenner et al., 2002). Furthermore, 763 CDSs could be annotated by the RAST subsystem, the top five coded subsystem functions are “Cofactors, Vitamins, Prosthetic Groups, Pigments (70),” “Protein Metabolism (138),” “DNA Metabolism (56),” “Amino Acids and Derivatives (151)” and “Carbohydrates (107),” and no CDS was annotated to “Nitrogen Metabolism or Denitrification” (Figure 1C); annotation using eggNOG-mapper produced similar results to that of the RAST, excluding “S: Functions unknown,” the top five COGs are “E: Amino acid transport and metabolism,” “G: Carbohydrate transport and metabolism,” “J: Translation, ribosomal structure, and biogenesis,” “K: Transcription” and “L: Replication, recombination, and repair” (Figure 1D).

### 3.3. Evidence for taxonomic reassignment of *W. neuii*

**Figure 2:**
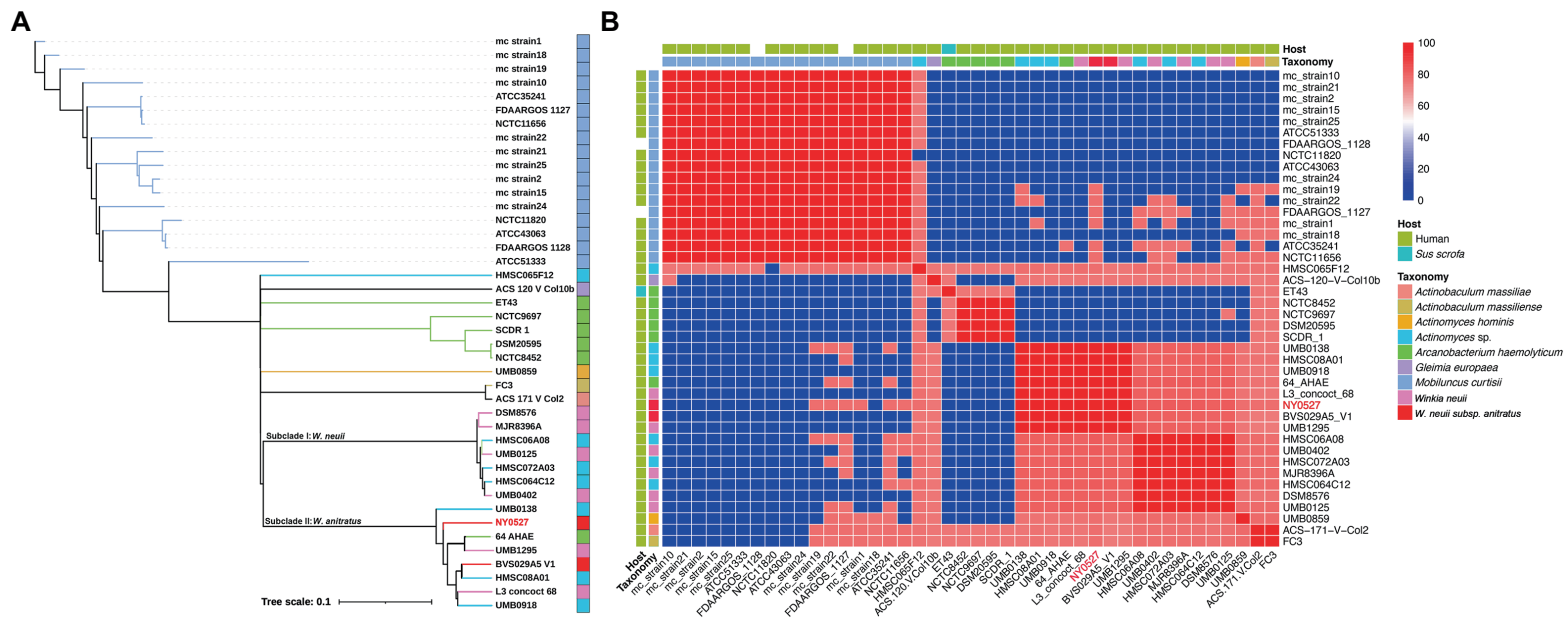
Phylogeny of strain NY0527 characterized by a phylogenomic tree and whole-genome-sequence-based ANI analysis. **(A)** WGS-based phylogenomic tree of phylogenetically close strains. Strain NY0727 is labeled in red. The taxonomy color code is shared with **(B)** in the legend. **(B)** Heatmap displaying the ANI between the phylogenetically close species. The color bar on the right indicates the ANI value calculated using fastANI.

To determine the phylogeny of strain NY0527 and to eliminate the divergence between the results of MALDI Biotyper and GTDB-tk, whole genome sequence based phylogenic tree and ANI analysis were performed. The Similar Genome Finder identified that genome sequences from eight species, namely *Mobiluncus curtisii*, *W. neuii*, *Arcanobacterium haemolyticum*, *Actinobaculum massiliae*, *Actinobaculum massiliense*, *Actinomyces hominis*, *Arcanobacterium haemolyticum*, and *Gleimia europaea*, as well as *Actinomyces* sp. are mostly close to that of strain NY0527 by genome distance (GD) (data not shown). All of the genomes from the eight species as well as the *Actinomyces* sp. genomes that were closely related to NY0527, were then downloaded from the NCBI genome RefSeq database, and fed to fastANI for ANI analysis, and their whole-genome CDSs were uploaded to CVTree3 for phylogenomic tree construction. A total of 42 genome sequences were collected, detailed information is provided in Supplementary Table S3. The GD clustering revealed that all of the *M. curtisii* genomes formed a divergent clade (i.e., clade I), separate from the others (i.e., clade II) (Supplementary Figure S1). Moreover, two subclades mixed by the *W. neuii* and *Actinomyces* sp. genomes, along with an *A. haemolyticum* genome, (64_AHAE) were formed within clade II, with a GD higher than 0.05 between the subclades, suggesting that *W. neuii* could be reclassified and separated into two species (Supplementary Figure S1). The phylogenomic tree displayed identical results to that of the GD clustering, which showed the formation of a divergent *M. curtisii* clade and the formation of two subclades consisting of 15 *W. neuii*, *Actinomyces* sp. and *A. haemolyticum* strains (Figure 2A). Except for *A. haemolyticum*, which was isolated from *Sus scrofa*, all others were isolated from human source (Figure 2B). The ANI heatmap determined that the divergence of these two subclades is at the species level, as the within-subclade ANI > 95%, while the trans-subclade ANI < 85% (Figure 2B). Furthermore, strain ET43 should be removed from *A. haemolyticum* and reassigned as a novel species; strains ACS-171-V-Col2 and FC3 from *A. massiliae* and *A. massiliense* should be merged as one species (Figures 2A,B). Collectively, the currently documented *W. neuii* strains should be divided into two separate species, one clustered with the type strain of *W. neuii* subsp. *neuii* DSM8576^T^, consisting of seven strains (i.e., DSM8576, MJR8396A, UMB0125, HMSC06A08, HMSC072A03, UMB0402, and HMSC064C12); another clustered with *W. neuii* subsp. *anitratus* strains and should be reassigned as a separate *Winkia* species (referred as *W. anitratus* below in the text), consisting of eight strains (i.e., NY0527, UMB1295, 64_AHAE, UMB0918, UMB0138, L3_concoct_68, HMSC08A01 and BVS029A5) (Figure 2B).

### 3.4. Pan-genomic features of *W. neuii* and *W. anitratus*

**Figure 3:**
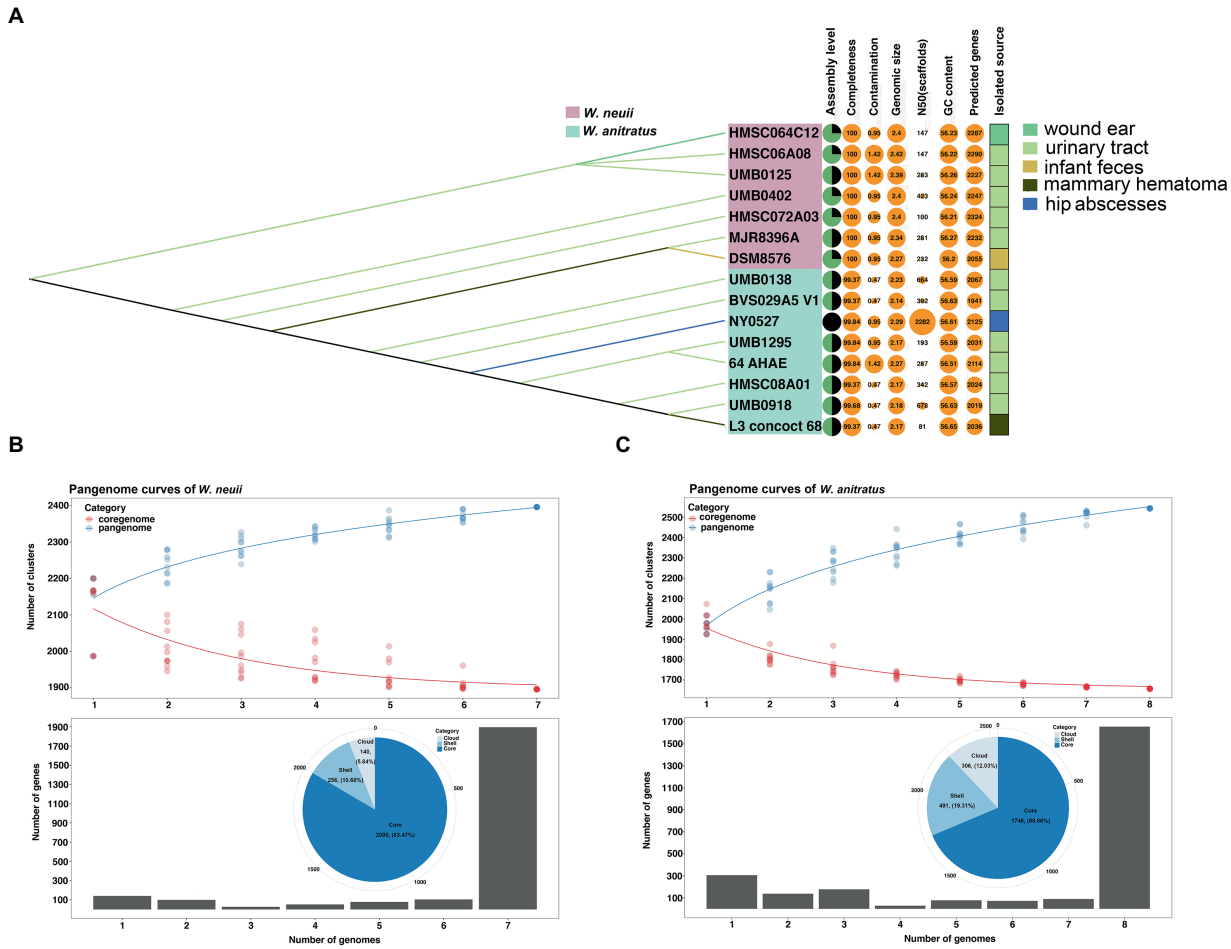
The comparison of pangenomic features between *W. neuii* and *W. anitratus* strains. **(A)** Characterization of the general genomic feature and isolation source. **(B,C)** Pangenomic features of *W. neuii* and *W. anitratus* strains.

Further characterization of the 15 *Winkia* genomes revealed that the genome of NY0527 is currently the sole genome that has been assembled at the completed level in the NCBI genome RefSeq database, the remaining genomes are at contigs or scaffolds levels (Figure 3A). The assemble qualities of these genomes are in high quality, with completeness greater than 99% and contaminations lower than 5%. Comparisons between the *W. anitratus* and *W. neuii* genome sequences showed that the *W. anitratus* has a lower genomic size (*W. anitratus*: 2.20 ± 0.05 Mb; *W. neuii*: 2.38 ± 0.05 Mb), a lower predicted gene counts (*W. anitratus*: 2044 ± 58; *W. neuii*: 2237 ± 88), but a higher G + C content (*W. anitratus*: 56.60 ± 0.04; *W. neuii*: 56.23 ± 0.03) than that of *W. neuii* (Figure 3A). As human pathogens, most of the *W. anitratus* (6/8) and *W. neuii* (5/7) strains that had genome sequenced were isolated from the infected urinary tracts. Others were isolated from wound ear, infant feces, mammary hematoma and hip abscesses (Figure 3A). Although the *W. neuii* had larger genome sizes and more predicted genes, pangenome analysis displayed that the *W. anitratus* strains carry much opener genomes, with higher count of pangenome clusters and lower count of coregenome clusters (Figures 3B,C).

### 3.5. Virulence factor coding capacity of *W. neuii* and *W. anitratus*

**Figure 4:**
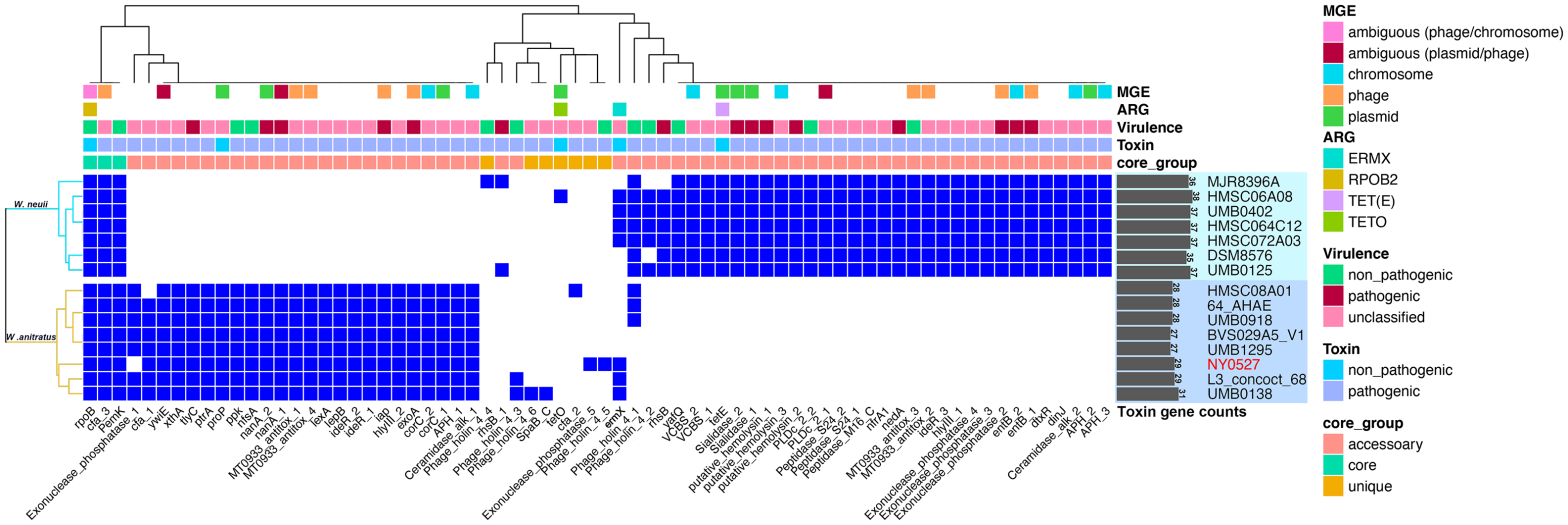
Pathogenic factor genes predicted in *W. neuii* and *W. anitratus* genomes. Hierarchical clustering was performed using the presence/absence pattern of the genes in the genomes. A blue cell in the heatmap indicates the presence of a gene. The virulence and toxin genes were defined as pathogenic when they were identified as pathogenic by both the HMM model and the random forest classifier.

To explore the potential pathogenies of *W. neuii* and *W. anitratus*, PathoFact was employed to predict the virulence factor coding genes in their pangenome. We found that *W. neuii* and *W. anitratus* displayed distinct profiles in the virulence factor coding genes, thus enabling a clear differentiation between them. Only three virulence factor coding genes, namely *rpoB*, *cfa_3*, and *pemK*, were shared by two species (Figure 4). Each *W. neuii* strain encoded 35–38 virulence factors, higher than that of *W. anitratus* strain, which encoded 27–31 virulence factors, indicating that *W. neuii* has a higher pathogenicity potential than *W. anitratus* (Figure 4). At the species level, most of the virulence factor coding genes were shared by all strains within-species (Figure 4). Moreover, less than half of the virulence factor coding genes in both species were predicted as MGE-originated, whith the majority of such genes arising from phage- and plasmid-MGE (Figure 4). Only three categories of ARGs were identified, two (i.e., *rpoB* and *ermX*) of which in both species, encoding rifampicin resistance and macrolides/lincosamides/streptogramins resistance, and one (i.e., *tetO* or *tetE*) exclusively in *W. neuii*, encoding tetracycline resistance (Figure 4). Furthermore, 15 of the predicted virulence factor coding genes in the pangenome were recognized to be pathogenic, among which 10 were exclusively encoded by *W. neuii* and 5 were exclusively encoded by *W. anitratus* (Figure 4). Although the pathogenic virulence factor coding genes of *W. neuii* and *W. anitratus* differ greatly in counts and encoded functions, some of their pathogenic effects may be similar by encoding homologue proteins such as putative hemolysin and sialidase, which are shared by all *Winkia* strains (Table 2). Apart from this, an iota toxin coding gene is uniquely encoded by all *W. anitratus* strains, while a probable enterotoxin B coding gene and an iron dependent repressor coding gene are uniquely carried by all *W. neuii* strains (Table 2). Interestingly, some of the *W. neuii* strains encode insecticidal toxin complex proteins (TccC), although they were isolated from human sources (Table 2).

**Table 2:** Characterization of the virulence factor coding genes in *Winkia* spp.

Name	Presence (%) (*n*/*n*)		MGE_prediction	Potential pathogenic effects
	*W. neuii*	*W*. *anitratus*		
*exoA*	0 (0/7)	100% (8/8)	Phage	exonuclease/phosphatase family, degradation of extracellular traps
*tlyC*	0 (0/7)	100% (8/8)	Unclassified	putative hemolysin C homolog, hemolytic activity to host cell
*iap*	0 (0/7)	100% (8/8)	Phage	iota toxin, a potent cytotoxin, destroy filamentous actin *via* mono-ADP-ribosylation of globular actin
*nanA_2*	0 (0/7)	100% (8/8)	Plasmid	Sialidase, facilitates their adhesion to host tissues by cleaving sialic acid residues from the host’s cell surface
*nanA_1*	0(0/7)	100% (8/8)	Plasmid or phage	Sialidase, facilitates their adhesion to host tissues by cleaving sialic acid residues from the host’s cell surface
Sialidase_1	100% (7/7)	0 (0/8)	Plasmid	Sialidase, facilitates their adhesion to host tissues by cleaving sialic acid residues from the host’s cell surface
Sialidase_2	100% (7/7)	0 (0/8)	Plasmid	Sialidase, facilitates their adhesion to host tissues by cleaving sialic acid residues from the host’s cell surface
*nedA*	100% (7/7)	0 (0/8)	Unclassified	Sialidase, facilitates their adhesion to host tissues by cleaving sialic acid residues from the host’s cell surface
hemolysin_1	100% (7/7)	0 (0/8)	Unclassified	putative hemolysin, hemolytic activity to host cell
hemolysin_2	100% (7/7)	0 (0/8)	Unclassified	putative hemolysin, hemolytic activity to host cell
*entB_1*	100% (7/7)	0 (0/8)	Chromosome	probable enterotoxin B, stimulation of cytokine release and inflammation
Exonuclease_phosdphatase_2	100% (7/7)	0 (0/8)	Unclassified	endonuclease/exonuclease/phosphatase family, degradation of extracellular traps
*entB_2*	100% (7/7)	0 (0/8)	Phage	probable enterotoxin B, stimulation of cytokine release and inflammation
*rhsB*	86% (6/7)	0 (0/8)	Unclassified	insecticidal toxin complex protein TccC
*rhsB_1*	29% (2/7)	0 (0/8)	Unclassified	insecticidal toxin complex protein TccC

## 4. Discussion

*W. neuii* has long been recognized as pathogenic to humans, and the types of infections it causes are increasingly observed with the development of advanced microbial identification systems. However, the taxonomy of this species has only recently been clarified, and its pathogenesis remains elusive. We isolated a clinically-derived *Winkia* strain NY0527 from the sanies of the hip abscess and assembled the complete genome sequences of the genus for the first time. We found that the infections could be effectively cured by using an antibiotic therapy of cefuroxime and ornidazole. We then determined that strain NY0527 is highly susceptible to the first-line clinical beta-lactam antibiotics. Further investigation disclosed that most of the *Winkia* spp. genomes carried the beta-lactamase expression repressor gene. Phylogenomic analyses and genome-based ANI analysis revealed that the two subspecies of *W. neuii* should be reclassified as two separate species (i.e., ANI < 95%). Moreover, we determined for the first time that the potent virulence factors of *Winkia* spp. are mainly plasmid- or phage-originated sialidase, hemolysin, and enterotoxins.

Abscesses are the most common human infections caused by *W. neuii*, which accounted for half of the reported cases (Zelyas et al., 2016). Fortunately, infections caused by *W. neuii* or related *Actinomyces* species usually have favorable outcomes after surgical debridement and antibiotic treatment (Könönen and Wade, 2015; Zelyas et al., 2016). In line with these previous observations, the patient infected with *W. anitratus* NY0527 was successfully treated after surgical debridement and a 14-day course of cefuroxime and ornidazole antibiotics. *Winkia* spp. had long been treated as commensals, as they were thought to only shift to pathogenesis in populations with iatrogenic interventions, myriad comorbidities, or immunodeficiency (Leal et al., 2016). In this study, however, the patient had no such medical history or immunodeficiency. This was also observed in a previous study that a patient got *W. neuii* associated neonatal sepsis but without immunodeficiency, anatomical abnormalities, or related diseases (Mann et al., 2002). These novel findings highlight the potential of *Winkia* spp. to achieve high pathogenicity under specific circumstances, which necessitates greater attention in clinical practice. Fortunately, *Winkia* spp. is generally highly sensitive to beta-lactam antibiotics (Mann et al., 2002; Graffi et al., 2012; Zelyas et al., 2016), as well as most other antibiotics, although it is sometimes resistant to aminoglycosides and fluoroquinolones (Hall and Copsey, 2015). In this study, the patient was successfully cured with a 14-day course of antibiotic therapy consisting of cefuroxime and ornidazole. The isolated strain *W. anitratus* NY0527 demonstrated susceptibility to most of the first-line clinical antibiotics (Table 1), which is in accordance with previous findings (Steininger and Willinger, 2016). However, a gene *ermX* that encodes resistance to macrolides, lincosamides, and streptogramins was carried by the plasmid of strain NY0527, which is flanked by an IS6 family transposase, and is likely responsible for the strain’s resistance to erythromycin and clindamycin (Figure 1; Table 1). Of note, the *ermX* gene may be carried by other *W. neuii* or *W. anitratus* strains (Figure 4), suggesting the possibility of acquired antibiotic resistance in *Winkia* spp., which should be highly noted in clinical settings. Despite carrying fluoroquinolone resistance genes on its chromosome, *W. anitratus* NY0527 is slightly susceptible to fluoroquinolone antibiotics (Table 1). In contrast, *W. anitratus* NY0527 is highly susceptible to tetracycline, and all of the *W. anitratus* strains carried no tetracycline resistant genes, whereas all of the *W. neuii* strains carried at least one tetracycline resistant gene (Figure 4). Furthermore, we found for the first time that 86.67% of the *Winkia* spp. genomes encode the beta-lactamase expression repressor gene (Supplementary Table S4). This suggests that beta-lactam antibiotics remain the preferred and safe choice for treating *Winkia* spp. infections in first-line clinical practice.

*Winkia* spp. had long been incorrectly classified as *Actinomyces* spp. due to the poorly resolved biochemical features and 16S rRNA-based phylogenetic trees (Nouioui et al., 2018). They were also often misidentified as skin contaminants, making accurate identification challenging (Gómez-Garcés et al., 2010). Only until recently, by using the genome-based taxonomic classification methods, such as the phylogenomic tree and pairwise genome-based ANI comparison, Nouioui et al. (2018) separated *A. neuii* from the genus *Actinomyces*, and reassigned it as a novel genus, namely *Winkia*. In this genus, *W. neuii* is currently the sole species validly published. These methods produced much better resolved taxonomic assignments than traditional approaches, which had been recognized as useful tools to further clarify organisms with agricultural, biotechnological and clinical importance (Chun et al., 2018; Ciufo et al., 2018; Nouioui et al., 2018). Our phylogenomic and ANI analyses showed that *W. neuii* was phylogenetically closer to *M. curtisii* than to the *Actinomyces* spp., and further clarified that the subspecies within *W. neuii* should be reclassified as two independent species (Figure 2). These two species can also be distinguished by general genomic features such as genome size, whole genome G + C content, and number of CDSs (Figure 3A). In recent years, the use of hybrid assembly techniques that combine long-reads sequencing technologies (e.g., Nanopore) with short-reads sequencing technologies (e.g., Illumina) has enabled the more efficient and accurate acquisition of complete genome sequences of clinically relevant microorganisms (Ben Khedher et al., 2022). These complete genome sequences serve as high-quality reference genomes for functional genomic analyses, such as investigations of virulence, antibiotic resistance, and mobile genetic elements (Ben Khedher et al., 2022). In our study, we recovered the first complete plasmid and chromosome sequences of *Winkia* spp. by utilizing this hybrid assembly approach. Our analysis of these sequences revealed potential recombination and transfer events occurring between *Winkia* spp. and their commonly observed co-colonizers, *Corynebacterium* spp. This suggests that gene exchange between different clinical bacterial genera should be considered. Disappointedly, the 15 WGS-sequenced *Winkia* spp. now published in the NCBI genome RefSeq database are isolated from very limited range of human body sites (e.g., wound ear, urinary tract, mammary hematoma, and buttock abscesses), despite the fact that they have been reported isolated from many other body sites, such as foreign body devices, the oral cavity, and blood (Yang and Grant, 2019). It is certain that more isolates from diverse body sites are required to be sequenced and completely assembled in the future to better comprehend their taxonomic diversities, pathogenesis and the gene exchange events with other co-colonized species.

Because *Winkia* spp. had not been isolated from non-human sources, as reported in previous studies and validated in this study, the infections caused by *Winkia* spp. are thought to be endogenous (von Graevenitz, 2011). Nevertheless, potential virulence factors carried by *Winkia* spp. have not been investigated thus far. We found that the *Winkia* spp. genomes encoded at least two core-pathogenic virulence factors (or their homologs), namely hemolysin and sialidase, to help them infect the host. Hemolysin has been proven to be a family of bacterial pore-forming toxin expressed in many pathogenic bacteria, including *Proteus*, *Morganella*, *E. coli* and *Moraxella* (Kaplan et al., 2009). For example, *E. coli* could produce hemolysin, which enables it to cause an infection in the urinary tract or other extraintestinal sites (Donnenberg, 2002). Numerous *Actinomyces* species produce sialidase, an enzyme that facilitates their adhesion to host tissues by cleaving sialic acid residues from the host’s cell surface. Sialidase also has the ability to deglycosylate immunoglobulins, thus altering the host’s immune response to the infection (Do et al., 2008; Bernard, 2012). Furthermore, some of the virulence factors are specifically encoded within-species between *W. neuii* and *W. anitratus.* For example, the iota toxin may produce cytotoxic activity through necrosis (Navarro et al., 2018) and is specifically coded by all *W. anitratus* strains, while the enterotoxin B may cause food poisoning with severe diarrhea and intestinal cramping (Bae et al., 2021), and is specifically coded by all *W. neuii* strains. Notably, the *W. neuii* encoded roughly twice as many pathogenic virulence factors as the *W. anitratus* (Figure 4), implying that the *W. neuii* is more virulent than the *W. anitratus*. This hypothesis can be corroborated by the fact that *W. neuii* subsp. *neuii* is more prevailed over *W. neuii* subsp. *anitratus* under clinical observations (von Graevenitz, 2011). As such, it is not surprising that the *Winkia* spp. can infect immune competent individuals in some cases. We therefore suggest strengthening the isolation, species identification, and whole genome sequencing of the clinically-derived *Winkia* spp. strains in the future, which would be useful in tailoring effective treatments or prevention schedules for the related infections.

In conclusion, we reported an infective hip abscess caused by a *Winkia* strain NY0527 and assembled the first completed genome sequences (i.e., one circular chromosome sequence and one circular plasmid sequence) of the genus *Winkia* for comparative genomic analysis. We found that the two subspecies of *W. neuii* could be reclassified as two separate species, and thereby emphasize the potential underestimation of taxonomic diversity within the genus. For the first time, we demonstrated the mechanisms of antibiotic resistance and pathogenesis of *Winkia* spp. at genus and species levels by using *in silico* genomic analyses. We further suggested that beta-lactam antibiotics can still be effectively utilized as first-line antibiotics for the treatment of *Winkia* spp. infections. The findings of this study provide a global clue of the pathogenic, antibiotic-resistant, and taxonomic features of *Winkia* spp. and underline the importance of focusing on emerging *Winkia* spp. infections and their potential antibiotic resistances.

## Data availability statement

The datasets presented in this study can be found in online repositories. The names of the repository/repositories and accession number(s) can be found at: https://www.ncbi.nlm.nih.gov/, PRJNA925112.

## Ethics statement

The studies involving human participants were reviewed and approved by the Ethics Committee of Shenzhen University General Hospital. The patients/participants provided their written informed consent to participate in this study.

## Author contributions

XC, YP, and ML performed the bacteria isolation and taxa identification. XC and YP performed the WGS-based analysis and drafted the manuscript. YQ and YW reviewed and revised the manuscript. LX and QH designed and supervised the whole study. All authors made substantial and direct contributions to the work, and read and approved the final version of the manuscript.

## Funding

This work was supported by the National Natural Science Foundation of China (41907214 and 82002716); the Shenzhen Science and Technology Program (JCYJ20190808111610984 and JCYJ20190808164209301); the Project of Department of Education of Guangdong Province (2019KTSCX146); Shenzhen Scientific Research Foundation for Excellent Returned Scholars (000493); Natural Science Foundation of Shenzhen University General Hospital (SUGH2020QD005); Shenzhen Key Laboratory Foundation (ZDSYS20200811143757022).

## Conflict of interest

The authors declare that the research was conducted in the absence of any commercial or financial relationships that could be construed as a potential conflict of interest.

## Publisher’s note

All claims expressed in this article are solely those of the authors and do not necessarily represent those of their affiliated organizations, or those of the publisher, the editors and the reviewers. Any product that may be evaluated in this article, or claim that may be made by its manufacturer, is not guaranteed or endorsed by the publisher.

## Footnotes

^2^ https://www.patricbrc.org/

^3^ https://github.com/leaemiliepradier/PlasForest

^4^ https://rast.nmpdr.org
