# Supplementary material for: Comparative genomic analyses of the clinically-derived Winkia strain NY0527: the reassignment of W. neuii subsp. neuii and W. neuii subsp. antitratus into two separate species and insights into their virulence characteristics
Source: Front Microbiol. 2023 Apr 21;14:1147469. doi: 10.3389/fmicb.2023.1147469 (PMC10160630; doi:10.3389/fmicb.2023.1147469)
Supplement: Supplementary file 1 [file Data_Sheet_1.PDF]

Comparative Genomic Analyses of the Clinically-Derived *Winkia* Strain NY0527: the Reassignment of *W. neuii* subsp. *neuii* and *W. neuii* subsp. *antitratus* into Two Separate Species and Insights into Their Virulence Characteristics

Xunchao Cai [a], Yao Peng [a], Meng Li [b], Yifeng Qiu [c], Yuhan Wang [c], Long Xu [a, *], Qi Hou [c, d, *]

[a] Department of Gastroenterology and Hepatology, Shenzhen University General Hospital, Shenzhen University, Shenzhen, China

[b] Department of Laboratory Medicine, Shenzhen University General Hospital, Shenzhen University, Shenzhen, China.

[c] Department of Urology, Shenzhen University General Hospital, Shenzhen University, Shenzhen, China

[d] International Cancer Center, Shenzhen Key Laboratory, Hematology Institution of Shenzhen University, Shenzhen, China

[*]Correspondence to:

Long Xu, Department of Gastroenterology and hepatology, Shenzhen University General Hospital, Shenzhen University, 518055, Shenzhen, China. Email: longxu1012@szu.edu.cn; Qi Hou, Department of Urology, Shenzhen University General Hospital, Shenzhen University, 518055, Shenzhen, China. Email: qi_hou@foxmail.com.

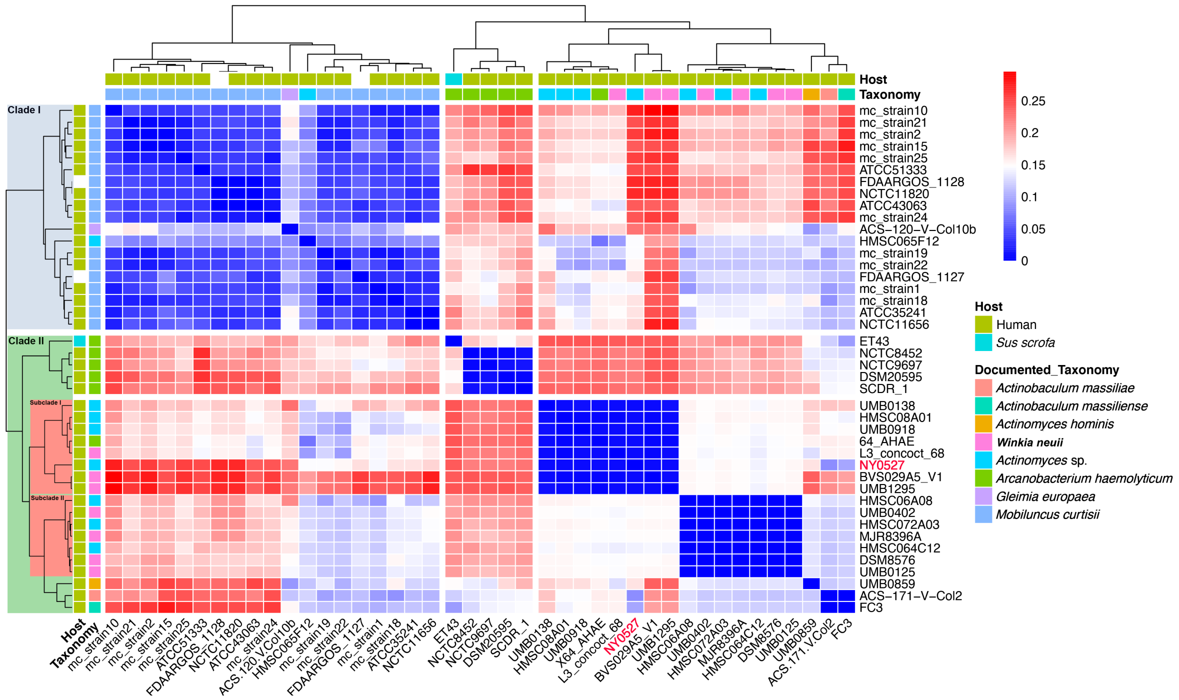

Figure S1 Heatmap displaying the genome distances between the strains phylogenomically close to strain NY0527.

Table S1 Taxonomic assignment of strain NY0527 using the MALDI Biotyper

Taxon	Strain	Score	Confidence Level
W. neuii subsp. *anitratus*	CIP104016T	2.171	Species
W. neuii subsp. *anitratus*	CCUG26929	2.161	Species
W. neuii subsp. *anitratus*	ENR_0422	2.057	Species
W. neuii	475RLT	2.051	Species
W. neuii	VA201_10 ERL	1.968	Genus
W. neuii	50_90 IBS	1.884	Genus
W. neuii subsp. *anitratus*	121 RLT	1.805	Genus
W. neuii	461 RLT	1.8	Genus
W. neuii subsp. *anitratus*	DSM 8577^{T}	1.581	Not significant
W. neuii subsp. *neuii*	DSM 8576^{T}	1.521	Not significant

Table S2 Sequence alignment of plasmid pNY0527 against the NCBI-nt database[a]

Taxonomy	Strain	Query Coverage	Identity	Accession	Sequence Type
Corynebacterium striatum	M82B	53%	99.93%	AF024666.2	Plasmid
Corynebacterium diphtheriae	FRC0137	44%	99.92%	OV884287.1	Chromosome
Corynebacterium diphtheriae	FRC0375	44%	99.92%	OV884286.1	Chromosome
Corynebacterium striatum	KC-Na-01	40%	99.92%	CP021252.1	Chromosome
Corynebacterium striatum	FDAARGOS_1115	41%	99.87%	CP068158.1	Chromosome
Corynebacterium diphtheriae	FRC0402	52%	99.74%	OV884290.1	Plasmid
Corynebacterium striatum	215	54%	99.47%	CP024931.1	Chromosome
Corynebacterium urealyticum	FDAARGOS_995	50%	99.06%	CP066289.1	Chromosome
Corynebacterium urealyticum	DSM 7111	50%	98.61%	CP004085.1	Chromosome
Corynebacterium urealyticum	FDAARGOS_996	40%	98.61%	CP065982.1	Chromosome
Corynebacterium urealyticum	FDAARGOS_994	50%	98.4%	CP066064.1	Chromosome
Corynebacterium urealyticum	NCTC12011	50%	98.33%	LT906481.1	Chromosome
Corynebacterium urealyticum	DSM 7109	50%	98.33%	AM942444.1	Chromosome
Corynebacterium kefirresidentii	FDAARGOS_1055	51%	95.69%	CP067011.1	Plasmid
Corynebacterium striatum	216	59%	95.43%	CP024932.1	Chromosome
Corynebacterium striatum	FDAARGOS_1197	59%	95.4%	CP069514.1	Chromosome
Corynebacterium striatum	KC-Na-01	40%	93.9%	CP021253.1	Plasmid

[a] Only sequences with query-coverage higher than 40% are listed here.

Table S3 Information of the genomes downloaded for analysis in this study

Strain	Taxonomy	Assembly accession /Nucleotide accession
64_AHAE	*Arcanobacterium haemolyticum*	GCA_001055535.1
BVS029A5_V1	*Winkia neuii* subsp. *anitratus*	GCA_000296485.1
UMB1295	*Winkia neuii*	JAAUWI010000010,JAAUWI010000011,JAAUWI010000012,JAAUWI010000013,JAAUWI010000014,JAAUWI010000015,JAAUWI010000016

UMB0918	*Actinomyces* sp.	GCA_002872055.1
L3_concoct_68	*Winkia neuii*	GCA_018372675.1
HMSC08A01	*Actinomyces* sp.	GCF_001807435.1
UMB0138	*Actinomyces* sp.	GCA_002849225.1
HMSC06A08	*Actinomyces* sp.	LWOG01000001.1-LWOG01000045.1
UMB0125	*Winkia neuii*	GCA_002860645.1
HMSC064C12	*Actinomyces* sp.	GCF_001812345.1
MJR8396A	*Winkia neuii*	GCF_001546135.1
HMSC072A03	*Actinomyces* sp.	GCF_001812065.1
UMB0402	*Winkia neuii*	GCA_002860625.1
DSM8576	*Winkia neuii* subsp. *neuii*	GCA_000420405.1
ACS-171-V-Col2	*Actinobaculum massiliae*	GCA_000315465.1
mc_strain19	*Mobiluncus curtisii*	GCA_012976495.1
mc_strain1	*Mobiluncus curtisii*	GCA_012976845.1
FDAARGOS_1127	*Mobiluncus curtisii*	GCA_016725225.1
NCTC11656	*Mobiluncus curtisii*	GCA_900453315.1
FC3	*Actinobaculum massiliense*	GCA_001457435.1
UMB0859	*Actinomyces hominis*	GCA_002871945.1
HMSC065F12	*Actinomyces* sp.	GCF_001814535.1
mc_strain18	*Mobiluncus curtisii*	GCA_012976525.1
ACS-120-V-Col10b	*Gleimia europaea*	GCF_000411155.1
ATCC35241	*Mobiluncus curtisii*	GCA_000146285.1
ATCC43063	*Mobiluncus curtisii*	GCA_000196535.1
ATCC51333	*Mobiluncus curtisii*	GCA_000185425.1
DSM20595	*Arcanobacterium haemolyticum*	GCA_000092365.1
ET43	*Arcanobacterium haemolyticum*	GCA_021532125.1
FDAARGOS_1128	*Mobiluncus curtisii*	GCA_016726965.1
mc_strain10	*Mobiluncus curtisii*	GCA_012976565.1
mc_strain15	*Mobiluncus curtisii*	GCA_012976515.1

mc_strain2	*Mobiluncus curtisii*	GCA_012976795.1
mc_strain21	*Mobiluncus curtisii*	GCA_012976485.1
mc_strain22	*Mobiluncus curtisii*	GCA_012976465.1
mc_strain24	*Mobiluncus curtisii*	GCA_012976435.1
mc_strain25	*Mobiluncus curtisii*	GCA_012976425.1
NCTC11820	*Mobiluncus curtisii*	GCA_900450555.1
NCTC8452	*Arcanobacterium haemolyticum*	GCA_900475915.1
NCTC9697	*Arcanobacterium haemolyticum*	GCA_900445275.1
NY0527	*Winkia neuii* subsp. *anitratus*	
SCDR_1	*Arcanobacterium haemolyticum*	GCA_006088775.1

Table S4 Presence of the four homologs of beta-lactamase expression repressor coding gene in *Winkia* spp.

Strain	Homolog1	Homolog2	Homolog3	Homolog4
64_AHAE	A	P	A	A
BVS029A5_V1	A	A	A	A
DSM8576	P	A	A	A
HMSC064C12	P	A	A	A
HMSC06A08	A	P	A	A
HMSC072A03	P	A	A	A
HMSC08A01	A	A	P	A
L3_concoct_68	A	P	A	A
MJR8396A	P	A	A	A
NY0527	A	A	A	P
UMB0125	P	A	A	A
UMB0138	A	P	A	A
UMB0402	P	A	A	A
UMB0918	A	A	P	A
UMB1295	A	A	A	A

A and P represent absence and presence, respectively.
